# Exogenous 2-(3,4-Dichlorophenoxy) triethylamine alleviates salinity stress in maize by enhancing photosynthetic capacity, improving water status and maintaining K^+^/Na^+^ homeostasis

**DOI:** 10.1186/s12870-020-02550-w

**Published:** 2020-07-23

**Authors:** Lijie Li, Wanrong Gu, Liguo Zhang, Congfeng Li, Xichang Chen, Chunrong Qian, Zhenhua Wang, Wenhua Li, Shiyu Zuo, Shi Wei

**Affiliations:** 1 College of Agriculture, Northeast Agricultural University, Harbin, 150030 P. R. China; 2 College of Life Science and Technology, Henan Institute of Science and Technology, Xinxiang, 453000 Henan P. R. China; 3 Institute of Maize Research, Heilongjiang Academy of Agricultural Sciences, Harbin, 150030 P. R. China; 4 Institute of Crop Science, Chinese Academy of Agricultural Sciences, Beijing, P. R. China

**Keywords:** DCPTA, Salinity stress, Photosynthesis, Water status, Ion homeostasis

## Abstract

**Background:**

Soil salinity restricts plant growth and productivity. 2-(3,4-dichlorophenoxy) triethylamine (DCPTA) can alleviate salinity stress in plants. However, the mechanism of DCPTA-mediated salinity tolerance has not been fully clarified. We aimed to investigate its role in enhancing photosynthetic capacity, improving water status, maintaining K^+^/Na^+^ homeostasis and alleviating salinity stress in maize (*Zea mays* L.).

**Results:**

In present study, maize seedlings were grown in nutrient solutions with a combination of NaCl (0, 150 mM) and DCPTA (0, 20, 100, and 400 μM). And photosynthesis, water status, ion homeostasis and the expression of genes involved in ion uptake and transport were evaluated in the maize seedlings. The results demonstrated that DCPTA alleviated the growth inhibition of maize seedlings exposed to salinity stress by increasing the net photosynthetic rate (P_n_) and the quantum efficiency of photosystem II (PSII) photochemistry. DCPTA improved the root hydraulic conductivity, which help maintained the water status. A relatively high K^+^ concentration but a relatively low Na^+^ concentration and the Na^+^/K^+^ ratio were observed in the presence of DCPTA under salinity stress. Additionally, DCPTA altered the expression of four genes (*ZmSOS1*, *ZmHKT1*, *ZmNHX1* and *ZmSKOR*) that encode membrane transport proteins responsible for K^+^/Na^+^ homeostasis.

**Conclusions:**

DCPTA improved the salinity tolerance of maize may be associated with enhanced photosynthetic capacity, maintenance of water status and altered expression of genes involved in ion uptake and transport.

## Background

Soil salinization is one of the most severe adverse environmental factors limiting agricultural development [1, 2]. Approximately 830 million hectares (ha) of land (approximately 20% of the cultivated land area worldwide) is impacted by soil salinization [3–5], and an annual worldwide loss of US$12–27.3 billion occurs due to lost crop production [6]. It is expected that in 2050, the global population will surpass 9.1 billion, necessitating another 70% increase in food production to ensure food security [7]. Soil salinization will be the major obstacle in the way of achieving this goal [8].

Soil salinity initiates complex responses to inhibit plant growth and physiological processes [4, 9]. Initially soil salinity is known to represses plant growth in the form of osmotic stress due to high salt concentration in root zones, which is then followed by ion deficiency or toxicity due to acytosol over-accumulation of Na^+^ and Cl^−^ [10, 11]. Osmotic stress impairs cell water relations, inhibits cell expansion and division, and reduces stomatal aperture and transpiration [12, 13]. Stomatal closure limits the diffusion of atmospheric CO_2_ to the site of carboxylation and causes the stomatal limitation of photosynthesis, consequently decreasing growth rates [13]. Salinity-induced decreases in photosynthetic efficiency are often associated with the inhibition of photosystem II (PSII), which plays a central role in light energy conversion and electron transport [14]. Hydraulic conductivity (Lp) refers to the ease with which water can flow from one location to another and therefore influences the rate of water movement [13]; Lp can be used to indicate the ability of plant roots to absorb water [15]. Under salinity stress, the maintenance of Lp is an essential part of the adaptation process that helps to restore plant growth [13].

During long-term exposure to salinity, plants undergo ionic stress, particularly due to sodium chloride, which causes plant nutritional imbalance and oxidative stress, with severe consequences for plant growth, development and survival [4, 10, 12, 16]. Excess Na^+^ is particularly deleterious to plants because it competes with K^+^ for metabolic processes required for K^+^, leading to enzyme inactivation, plant nutritional imbalance, protein degradation, leaf photochemistry inhibition and oxidative stress [17]. All of these effects synergistically inhibit plant growth and development [10]. Previous studies have demonstrated that the K^+^/Na^+^ ratio is considered an important indicator for evaluating the salinity resistance of various plant species [4, 18]. The regulation of several genes encoding membrane transport proteins involved in Na^+^ and/or K^+^ uptake, translocation or compartmentalization is an important strategy for plants to address excessive Na^+^ accumulation and K^+^ deficiency. The plasma membrane Na^+^/H^+^ antiporter (*SOS1*, located in the plasma membrane) and the tonoplast (Na^+^, K^+^)/H^+^ antiporter (NHX1, located in the vacuolar membrane) can sequester Na + into vacuoles and play a major role in regulating cellular pH and Na^+^ homeostasis [9, 19]. The high-affinity potassium transporter 1 (*HKT1*) is involved in the control of Na^+^ long distance transport by reabsorption of Na^+^ from the xylem sap into the root cells, preventing the large accumulation of Na^+^ in the above-ground tissue [20, 21]. Moreover, the outward-rectifying K^+^ channel (*SKOR*) mediates K^+^ secretion from root cells into the xylem and K^+^ transport to the shoots [22].

The use of biostimulants, which are kinds of natural or synthetic small bioactive molecules derived from human- or animal-related industrial processes, is considered an effective measure to ameliorate growth inhibition induced by salinity or to improve plant resistance to salinity stress [10, 23]. The tertiary amine bioregulator 2-(3,4-dichlorophenoxy) triethylamine (DCPTA) represents a class of highly bioactive, low-molecular-weight amine compounds that have a significant regulatory effect on crop growth and development [24]. Previous studies have shown that DCPTA can improve the dry weight (DW) of tomato plants [25]; increase the root DW and leaf area of beet, radish [26], and maize [27]; and enhance ribulose-1,5-bisphosphate activity and increase the size of chloroplasts in the leaves of sugar beet (*Beta vulgaris* L.) [28]. DCPTA can also enhance CO_2_ fixation in cotton, promote chlorophyll biosynthesis in guayule [29] and stimulate carotenoid biosynthesis in citrus [30]. Suitable concentrations of DCPTA can improve the net photosynthetic rate (P_n_) in maize (*Zea mays* L.) [31] and have anti-senescent properties, as demonstrated by the slowing of chlorophyll degradation in bean (*Phaseolus vulgaris* L.) leaf discs in darkness [32]. Moreover, DCPTA can increase the ability of crops to adapt to stress and improve stress resistance [33–36]. Xie et al. [35] showed that spraying DCPTA can increase the leaf relative water content (LRWC) and promote water uptake, as indicated by increased Lp, which may be due to improvements in root growth, increased photosynthetic capacity associated with the increased chlorophyll content, greater photosynthetic C_4_ enzyme activity and reduced damage to chloroplasts under drought stress, thereby increasing the drought tolerance of maize seedlings. DCPTA can also increase tolerance to low-temperature stress by increasing the maximum quantum efficiency of PSII photochemistry (*F*_v_/*F*_m_) and the chlorophyll content, which effectively was shown to protect the photosynthetic system of maize leaves under low-temperature stress [33].

Maize is one of the most important cereal food crop species grown worldwide and provides raw materials for industry [37]. The area of maize production is the largest of food crop species, and its main planting areas are in irrigated agricultural areas in both arid and semi-arid regions. However, intensive irrigation results in high salinity levels [38]. The salinity-induced disturbance of nutritional status in growing cells inhibits maize growth and ultimately yield [39]. As maize is considered a moderately salinity-sensitive plant species [40], few salinity-tolerant maize cultivars have been commercialized [41]. Therefore, soil salinity stress has become one of the most serious threats to sustainable maize production [40]. A previous study showed that addition of DCPTA can alleviate reductions in root DW caused by salinity stress [34]. However, little is known about how DCPTA applications and doses can trigger adaptation to NaCl stress via morpho-physiological responses and ion homeostasis, particularly the molecular mechanisms involved. Therefore, the aims of this study were the following: (1) to determine the effects of DCPTA on the leaf photosynthetic capacity and chlorophyll fluorescence of maize exposed to salinity stress, (2) to evaluate the responses of the leaf water status (leaf water potential (Ψω) and LRWC) and Lp of maize in response to applications of DCPTA under salinity stress, and (3) to explore the regulation of the concentration of Na^+^ and K^+^ and the Na^+^/K^+^ ratio in the leaves and roots of maize and the expression of four genes (*ZmSOS1*, *ZmHKT1*, *ZmNHX1*, and *ZmSKOR*) that encode transport proteins responsible for Na^+^ and/or K^+^ uptake, translocation or compartmentalization in response to DCPTA in the leaves and roots of maize subjected to salinity stress. This systematic investigation will provide additional information for an improved understanding of the regulatory mechanisms of DCPTA-mediated salinity stress tolerance in maize.

## Results

### Exogenous DCPTA alleviates salinity-induced plant growth and biomass accumulation reductions

**Fig. 1:**
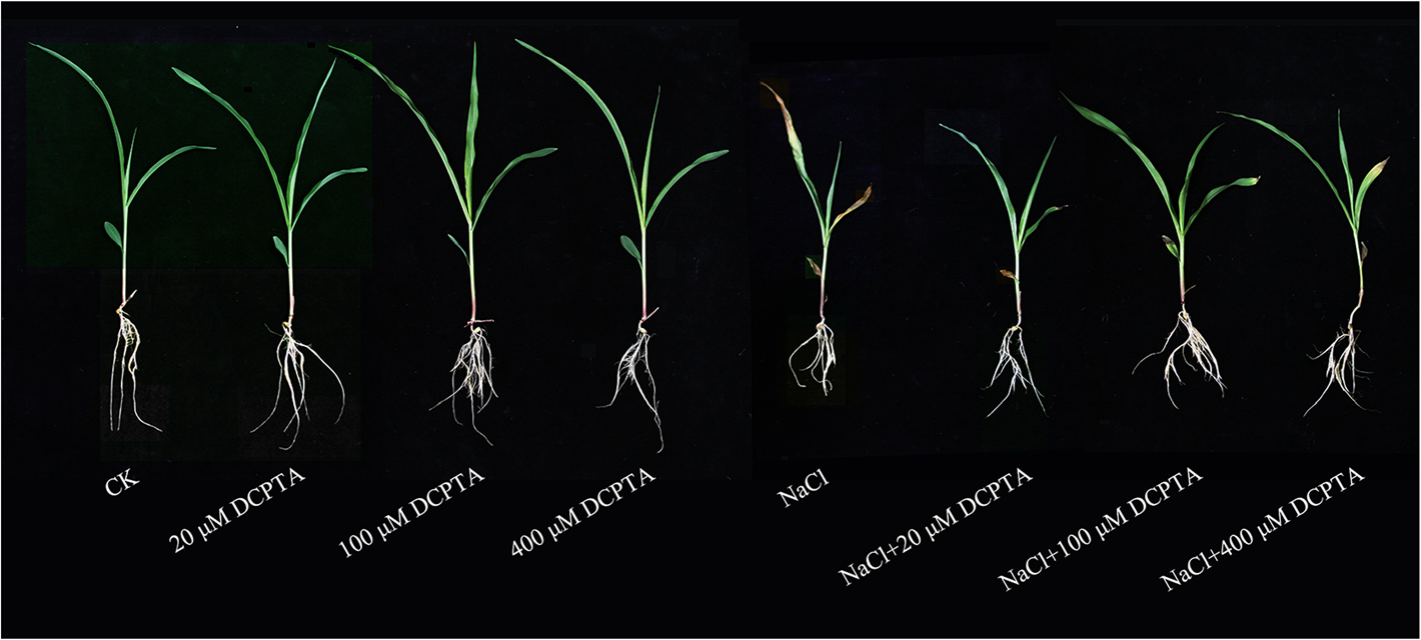
Effects of DCPTA on the growth performance of non-stressed or salinity-stressed maize plants. The plants were grown in a hydroponic solution that contained 0, 20, 100, or 400 μM DCPTA with or without 150 mM NaCl

**Fig. 2:**
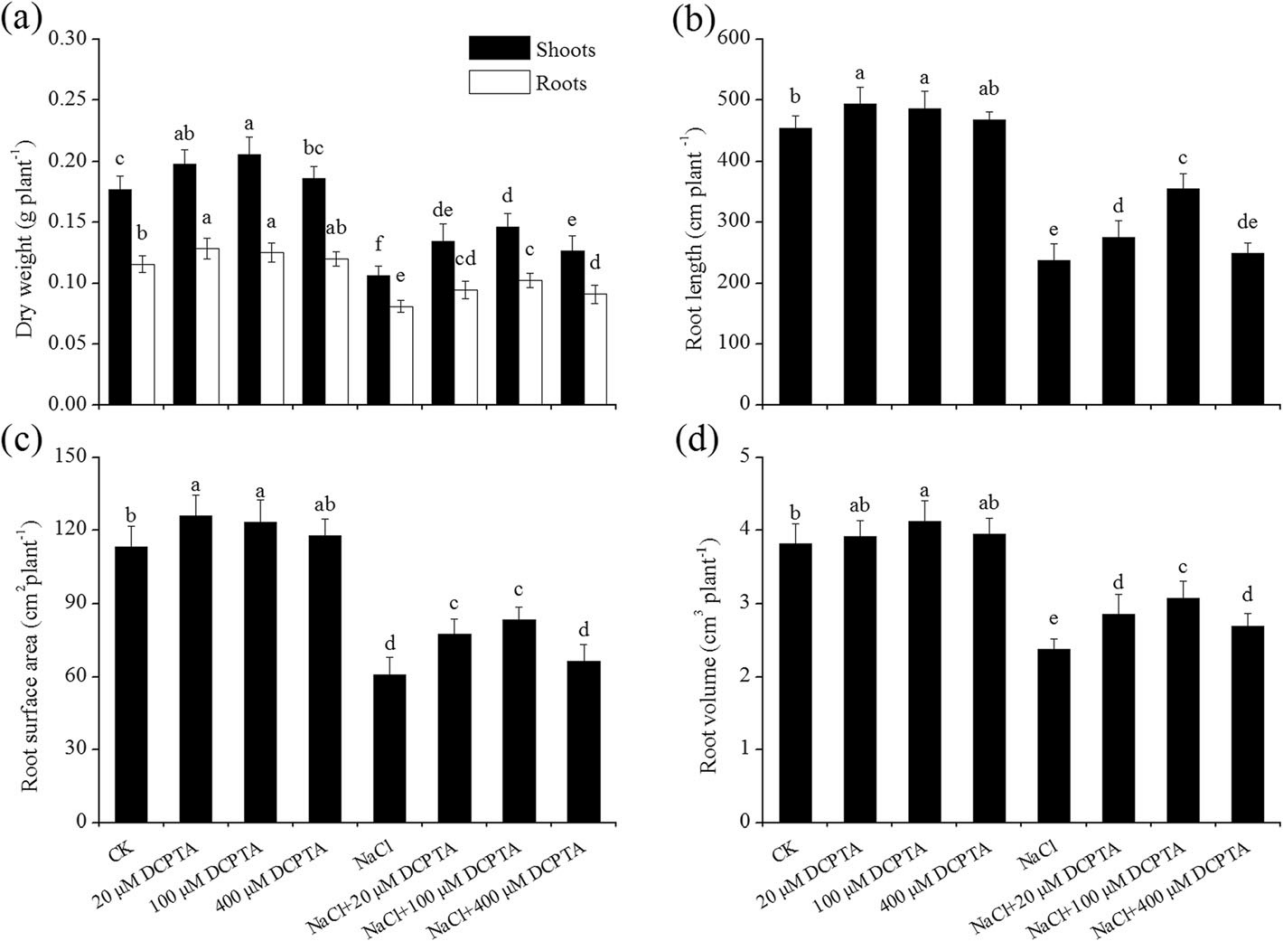
Effects of DCPTA on the DWs of shoot and roots (**a**), root length (**b**), root surface area (**c**), and root volume (**d**) of non-stressed and salinity-stressed maize plants. The plants were grown in a hydroponic solution that contained 0, 20, 100, or 400 μM DCPTA with or without 150 mM NaCl. The data are the means ± SEs (*n* = 5). The different letters on the bars indicate significant differences according to Duncan’s test (*P* = 0.05)

The DWs of root and shoot, and root length, surface area and volume of maize were significantly affected by NaCl and DCPTA (*P* ≤ 0.001), and the interaction between NaCl and DCPTA application rate had significant effect on the root length (Table 1). As shown in Fig. 1 and Fig. 2, under non-stressed conditions, low doses of DCPTA (20 μM and 100 μM) significantly increased the DWs of root and shoot, and the root length, surface area and volume, while a relatively high concentration of DCPTA (400 μM) had no significant effect on these indicators. Compared with non-salinity conditions, salinity stress significantly inhibited plant growth (*P* ≤ 0.05), and the DWs of root and shoot, and the root length, surface area and volume were reduced by 40.0, 30.1, 47.7, 46.4 and 37.5%, respectively. However, NaCl-induced reduction in the DWs of root and shoot and root volume were alleviated by applications of DCPTA at 20–400 μM, and the decreases in root length and root surface area were alleviated by applications of DCPTA at 20 and 100 μM, whereas 100 μM DCPTA appeared to be the most effective concentration for alleviating salinity stress. Under salinity-stressed conditions, compared with those of untreated maize, the DWs of root and shoot, and the root length, surface area and volume of maize treated with 100 μM DCPTA significantly improved by 33.4, 26.2, 39.4, 37.2 and 29.2%, respectively.

**Table 1:** Analysis of variance and mean comparisons for plant dry weight (Shoot DW: shoot dry weight; Root DW: root dry weight), leaf water status (RWC: relative water content; Ψw: leaf water potential), root hydraulic conductance (Lp) of maize plants grown under two salinity levels and treated with DCPTA at four rates of application

Source of variation	Shoot DW (g·plant ^− 1^)	Root DW (g·plant ^− 1^)	Root length (cm·plant ^− 1^)	Root surface area (cm^2^·plant ^− 1^)	Root volume (cm^3^·plant ^− 1^)	Ψw (MPa)	RWC (%)	Lp (10^−8^·m^3^·m^− 2^·s^− 1^ MPa^− 1^)
NaCl (mM) (N)								
0	0.19 ± 0.02a	0.12 ± 0.01a	475.64 ± 26.49a	120.12 ± 9.24a	3.95 ± 0.26a	−0.73 ± 0.06a	93.34 ± 2.43a	11.63 ± 0.75a
150	0.13 ± 0.02b	0.09 ± 0.01b	279.30 ± 52.22b	71.97 ± 10.93b	2.68 ± 0.32b	−1.69 ± 0.19b	71.99 ± 6.25b	6.00 ± 1.18b
DCPTA (μM) (D)								
0	0.14 ± 0.04b	0.10 ± 0.02a	346.20 ± 116.42a	86.95 ± 28.69a	3.10 ± 0.78a	−1.28 ± 0.62a	78.38 ± 15.69a	8.03 ± 3.45a
20	0.17 ± 0.04ab	0.11 ± 0.02a	384.99 ± 118.35a	101.75 ± 26.51a	3.24 ± 0.75a	−1.24 ± 0.58a	84.52 ± 10.90a	8.60 ± 3.22a
100	0.18 ± 0.03a	0.11 ± 0.02a	420.48 ± 73.39a	103.32 ± 22.29a	3.60 ± 0.60a	−1.09 ± 0.34a	85.38 ± 8.60a	9.89 ± 2.42a
400	0.16 ± 0.03ab	0.11 ± 0.02a	358.22 ± 116.24a	92.16 ± 27.96a	3.32 ± 0.69a	−1.23 ± 0.50a	82.35 ± 11.40a	8.74 ± 3.01a
Significance								
NaCl (mM) (N)	***	***	***	***	***	***	***	***
DCPTA (μM) (D)	***	***	***	***	***	***	***	***
N × D	ns	ns	**	ns	ns	**	**	*

ns, *, **, and ***: Not significant or significant at *P* ≤ 0.05, 0.01, and 0.001, respectively. The different letters within each column indicate significant differences according to Duncan’s multiple-range test (*P* = 0.05)

### Exogenous DCPTA maintains LRWC, leaf Ψω and root Lp

**Fig. 3:**
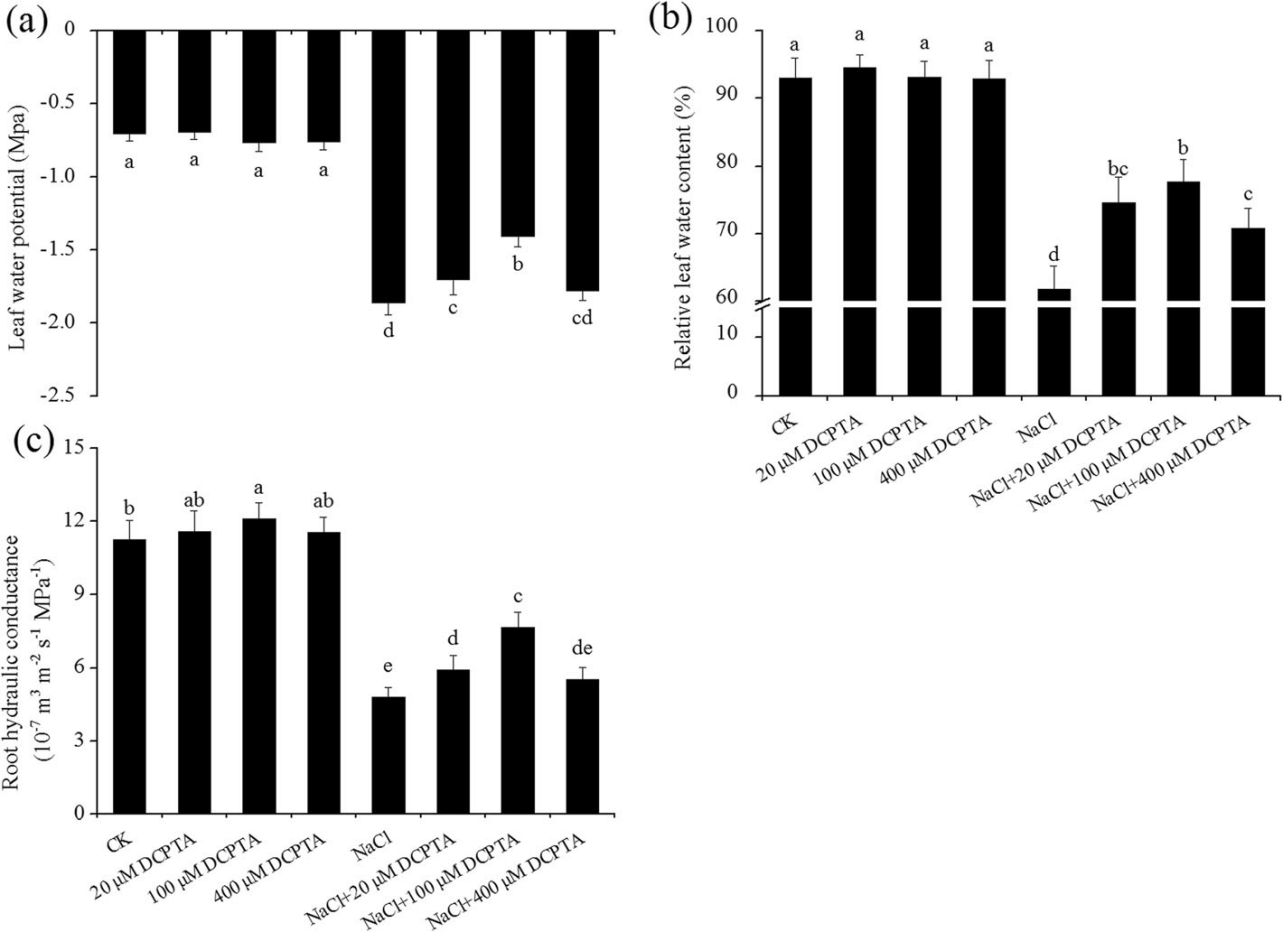
Effects of DCPTA on the leaf Ψω (**a**), LRWC (**b**), and root Lp (**c**) of non-stressed and salinity-stressed maize plants. The plants were grown in a hydroponic solution that contained 0, 20, 100, or 400 μM DCPTA with or without 150 mM NaCl. The data are the means ± SEs (*n* = 5). The different letters on the bars indicate significant differences according to Duncan’s test (*P* = 0.05)

The LRWC, leaf Ψω and root Lp were significantly affected by DCPTA and NaCl concentration; the interaction between DCPTA and NaCl concentration was significant (Table 1). Under the non-salinity conditions, application of DCPTA had no significant effect on leaf Ψω or LRWC; however, compared with the control, 100 μM DCPTA stimulated significant increases in Lp, which increased by 14.7% (Fig. 3). Compared with the non-stressed conditions, the salinity stress reduced the leaf Ψω, LRWC and Lp by 76.43, 45.56 and 57.4%, respectively. The NaCl-induced decreases in leaf Ψω and Lp were alleviated in plants treated with DCPTA at 20 μM and 100 μM – more markedly with the latter; however, the reduction in LRWC was alleviated in plants treated with DCPTA (20–400 μM), especially with DCPTA at 100 μM. Compared with salinity stress alone, the application of 100 μM DCPTA increased the leaf Ψω, LRWC and Lp by 24.4, 21.6 and 59.8%, respectively.

### Exogenous DCPTA improves photosynthetic pigment content and enhances photosynthetic capacity

**Fig. 4:**
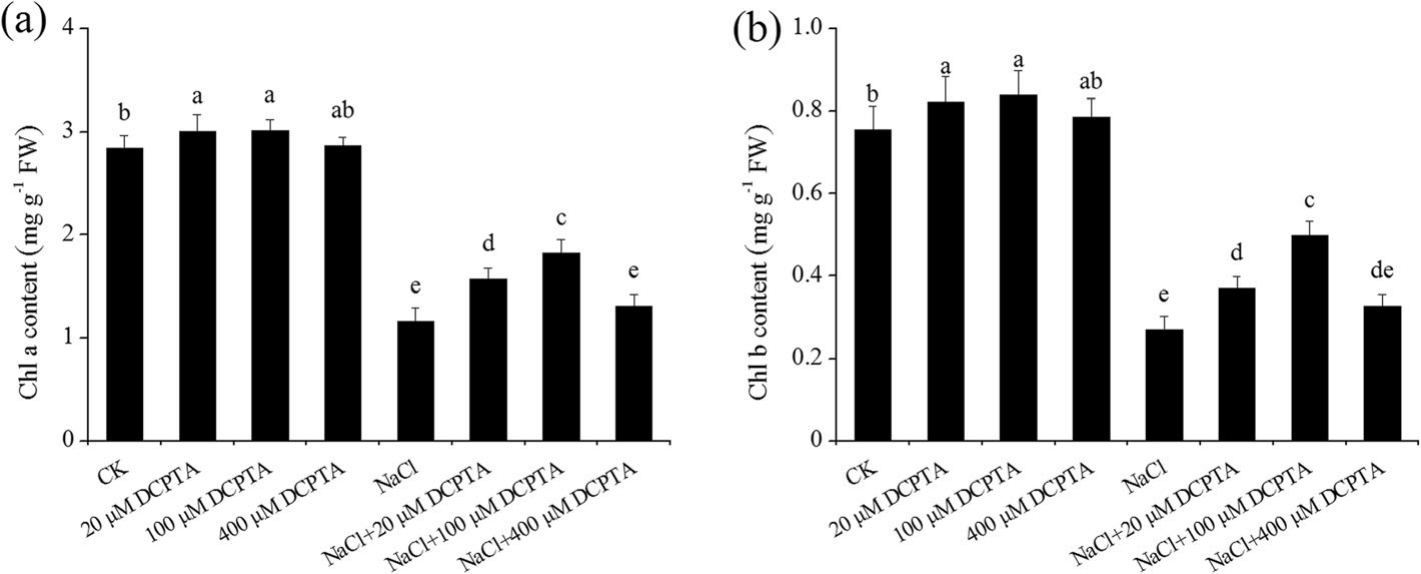
Effects of DCPTA on the Chl a content (**a**) and Chl b content (**b**) in the leaves of non-stressed and salinity-stressed maize seedlings. The plants were grown in a hydroponic solution that contained 0, 20, 100, or 400 μM DCPTA with or without 150 mM NaCl. The data are the means ± SEs (*n* = 5). The different letters on the bars indicate significant differences according to Duncan’s test (*P* = 0.05)

The contents of Chl a and Chl b were significantly affected by DCPTA and NaCl concentration, and the interaction between NaCl and DCPTA significantly affected the contents of Chl a and Chl b (Table 2). Under the non-stressed conditions, applications of DCPTA at 20 μM and 100 μM stimulated significant increases in the contents of Chl a and Chl b, while 400 μM DCPTA had no significant effect on chlorophyll content (Fig. 4). Compared with the non-stressed conditions, NaCl significantly affected Chl a and Chl b in maize and reduced their contents by 59.0 and 64.1%, respectively. Under salinity stress conditions, the 20 μM and 100 μM DCPTA-treated plants presented a greater increase in the contents of both Chl a and Chl b than did the plants not treated with DCPTA, and the increase was greater with 100 μM than with 20 μM DCPTA. Compared with salinity stress alone, 100 μM DCPTA increased the contents of Chl a and Chl b by 56.8 and 84.3%, respectively.

**Table 2:** Analysis of variance and mean comparisons for chlorophyll content (Chl a: chlorophyll a; Chl b: chlorophyll b), photosynthetic parameters (P_n_: net photosynthesis rate; g_s_: stomatal conductance; C_i_: intercellular CO_2_ concentration; T_r_: transpiration rate), chlorophyll fluorescence parameters (F_v_/F_m_: the maximum quantum efficiency of PSII photochemistry; ΦPSII: PSII operating efficiency; qP: photochemical quenching coefficient; NPQ: non-photochemical quenching) of maize plants grown under two salinity levels and treated with DCPTA at four rates of application

Source of variation	Chl a content (mg·g^−1^ FW)	Chl b content (mg·g^−1^ FW)	P_n_ (μmol·m^−2^·s^− 1^)	g_s_ (mmol·m^−2^·s^− 1^)	T_r_ (mmol·m^−2^·s^− 1^)	C_i_ (μmol·mol^− 1^)	*F*_v_/*F*_m_	ΦPSII	qP	NPQ
NaCl (mM) (N)										
0	2.93 ± 0.14a	0.80 ± 0.06a	15.05 ± 1.05a	0.08 ± 0.01a	4.67 ± 0.37a	224.69 ± 21.53b	0.83 ± 0.02a	0.74 ± 0.03a	0.64 ± 0.04a	0.57 ± 0.05b
150	1.47 ± 0.28b	0.37 ± 0.09b	9.11 ± 1.43b	0.05 ± 0.01b	2.30 ± 0.66b	337.81 ± 46.68a	0.58 ± 0.07b	0.39 ± 0.07b	0.40 ± 0.06b	0.81 ± 0.08a
DCPTA (μM) (D)										
0	2.00 ± 0.89a	0.51 ± 0.26a	11.00 ± 3.50a	0.06 ± 0.02a	3.13 ± 1.61a	298.90 ± 81.05a	0.67 ± 0.17a	0.52 ± 0.21a	0.48 ± 0.17a	0.75 ± 0.17a
20	2.29 ± 0.76a	0.60 ± 0.24a	12.30 ± 3.34a	0.07 ± 0.02a	3.62 ± 1.34a	283.03 ± 71.21a	0.69 ± 0.15a	0.56 ± 0.22a	0.54 ± 0.13a	0.68 ± 0.17a
100	2.42 ± 0.64a	0.67 ± 0.19a	13.50 ± 2.90a	0.07 ± 0.01a	3.87 ± 0.73a	251.35 ± 29.61a	0.74 ± 0.08a	0.60 ± 0.14a	0.54 ± 0.08a	0.65 ± 0.08a
400	2.09 ± 0.83a	0.56 ± 0.24a	11.52 ± 3.18a	0.06 ± 0.02a	3.32 ± 0.47a	291.72 ± 76.25a	0.72 ± 0.13a	0.58 ± 0.19a	0.50 ± 0.15a	0.68 ± 0.13a
Significance										
NaCl (mM) (N)	***	***	***	***	***	***	***	***	***	***
DCPTA (μM) (D)	***	***	***	***	***	***	***	***	***	**
N × D	**	**	**	ns	ns	ns	**	**	**	*

ns, *, **, and ***: Not significant or significant at *P* ≤ 0.05, 0.01, and 0.001, respectively. The different letters within each column indicate significant differences according to Duncan’s multiple-range test (*P* = 0.05)

**Fig. 5:**
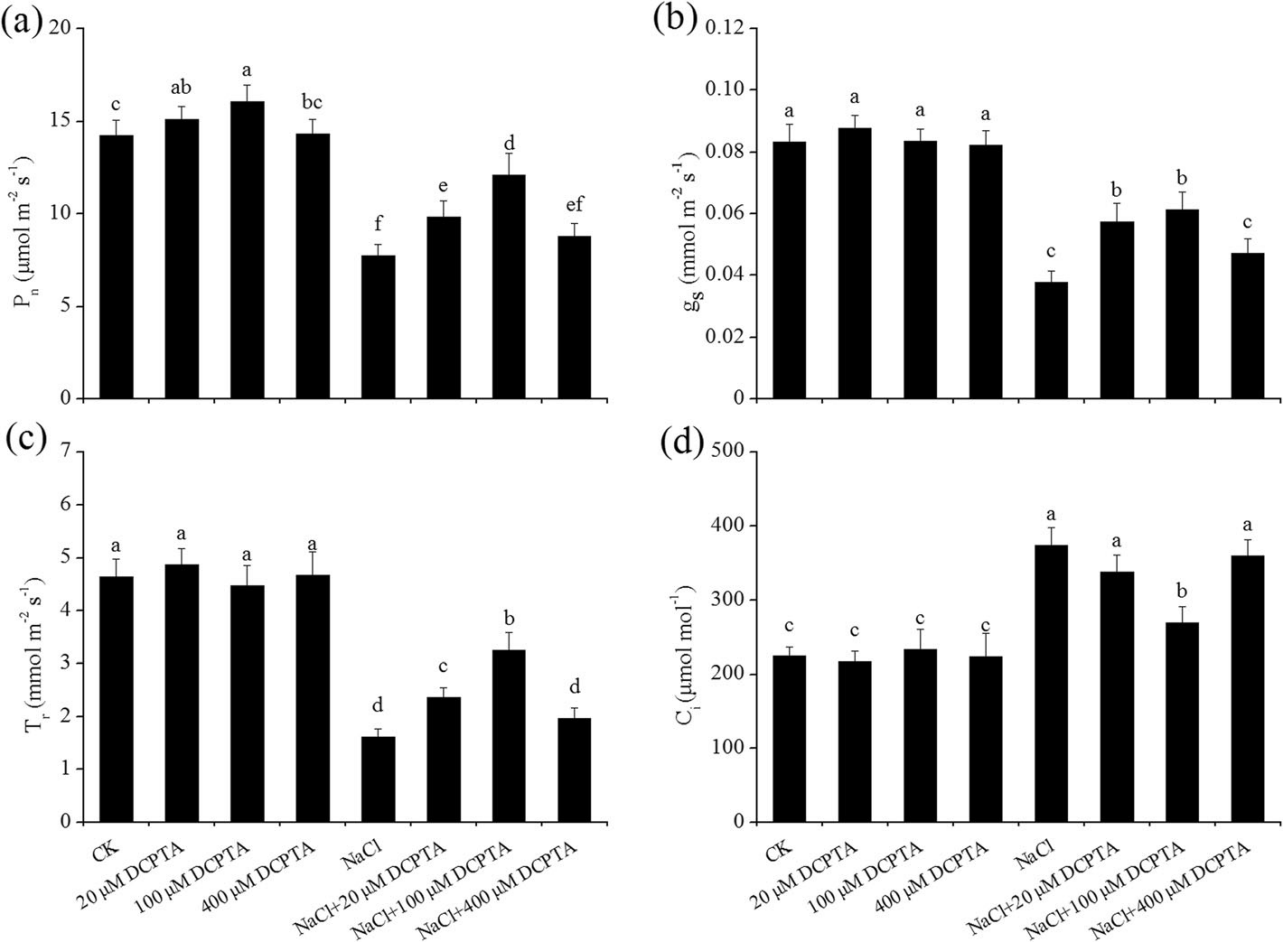
Effects of DCPTA on the P_n_ (**a**), g_s_ (**b**), T_r_ (**c**) and C_i_ (**d**), of the leaves of non-stressed and salinity-stressed maize seedlings. The plants were grown in a hydroponic solution that contained 0, 20, 100, or 400 μM DCPTA with or without 150 mM NaCl. The data are the means ± SEs (*n* = 5). The different letters on the bars indicate significant differences according to Duncan’s test (*P* = 0.05)

The P_n_, stomatal conductance (g_s_), intercellular CO_2_ concentration (C_i_) and transpiration rate (T_r_) were significantly affected by DCPTA and NaCl concentration. The interactive effects of DCPTA and NaCl concentration on the P_n_ were significant (Table 2). Under non-stressed conditions, low doses of DCPTA (20 μM and 100 μM) significantly increased the P_n_, while a relatively high concentration of DCPTA (400 μM) had no significant effect on P_n_ (Fig. 5a). The P_n_ increased by 7.9 and 12.9% in plants treated with 20 μM and 100 μM DCPTA, respectively, compared with the plants treated without DCPTA under non-stressed conditions. NaCl markedly decreased the P_n_ in maize leaves. Under stressed conditions, compared with that in non-DCPTA-treated plants, the P_n_ in plants treated with DCPTA at concentrations of 20 μM and 100 μM increased by 19.1 and 40.7%, respectively. Similarly, g_s_ and T_r_ were significantly reduced by salinity stress, and applications of DCPTA at 20 μM and 100 μM alleviated these adverse effects in the leaves of plants under stressed conditions (Fig. 5b and c). In contrast, salinity stress significantly stimulated an increase in C_i_, but this induction was attenuated by pre-treatment with DCPTA at 100 μM (Fig. 5d).

**Fig. 6:**
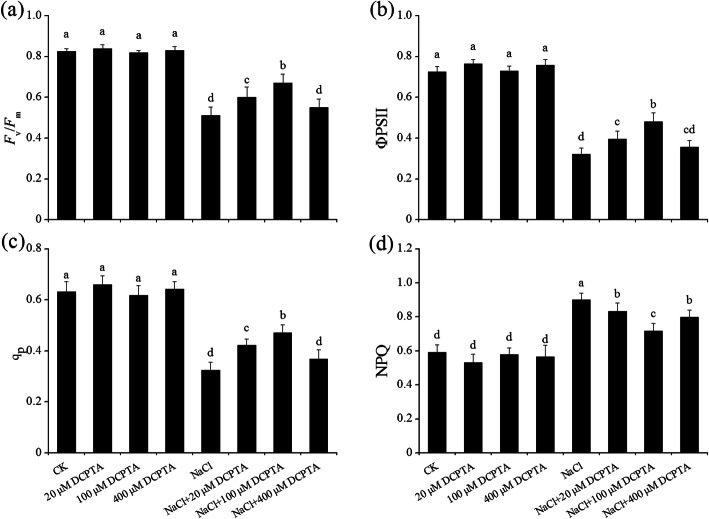
Effects of DCPTA on the *F*_v_/*F*_m_ (**a**), ΦPSII (**b**), qP (**c**), and NPQ (**d**) of the leaves of non-stressed and salinity-stressed maize seedlings. The plants were grown in a hydroponic solution that contained 0, 20, 100, or 400 μM DCPTA with or without 150 mM NaCl. The data are the means ± SEs (*n* = 5). The different letters on the bars indicate significant differences according to Duncan’s test (P = 0.05)

The *F*_v_/*F*_m_, ΦPSII, qP and NPQ were significantly affected by DCPTA and NaCl concentration. The interactions between DCPTA and NaCl concentration were significant (Table 2). Under non-stressed conditions, there was no effect of DCPTA on the obtained chlorophyll fluorescence parameters (Fig. 6). Compared with those in the control, the *F*_v_/*F*_m_, ΦPSII and qP were markedly declined, and the NPQ was significantly increased in response to treatment with 150 mM NaCl. The NaCl-induced decreases in *F*_v_/*F*_m_ and ΦPSII were alleviated by 100 μM and 400 μM DCPTA, especially with the former, while the decreases in qP were alleviated by applications of DCPTA at 20 μM and 100 μM – more markedly with the latter. NaCl-induced increase in NPQ was attenuated by pre-treatment with DCPTA (20–400 μM), and the effect of DCPTA was greater at 100 μM than at 20 μM and 400 μM. Compared with the salinity stress treatment, the DCPTA treatment at 100 μM markedly increased the *F*_v_/*F*_m_ by 31.1%, the ΦPSII by 49.5% and the qP by 45.2% and decreased the NPQ by 20.1%.

### Exogenous DCPTA attenuates Na^+^ toxicity, modulates Na^+^ and K^+^ homeostasis

**Fig. 7:**
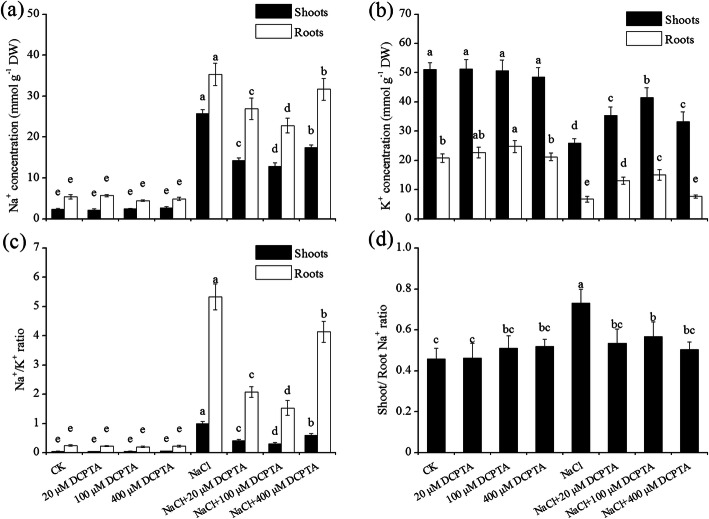
Effects of DCPTA on the concentrations of Na^+^ (**a**) and K^+^ (**b**) and on the Na^+^/K^+^ ratio (**c**) in the leaves and roots and the shoot/root Na^+^ ratio (**d**) in non-stressed and salinity-stressed maize seedlings. The plants were grown in a hydroponic solution that contained 0, 20, 100, or 400 μM DCPTA with or without 150 mM NaCl. The data are the means ± SEs (*n* = 5). The different letters on the bars indicate significant differences according to Duncan’s test (*P* = 0.05)

NaCl and DCPTA significantly affected the contents of Na^+^ and K^+^ and the Na^+^/K^+^ ratio in both the shoots and roots of maize plants (*P* ≤ 0.001). In addition, the interaction between NaCl and DCPTA significantly affected the contents of Na^+^ and K^+^ in both the shoots and roots, and the Na^+^/K^+^ ratio in the roots of maize plants (Table 3). As shown in Fig. 7, 150 mM NaCl significantly affected the concentrations of Na^+^ and K^+^ in both the roots and shoots of maize plants. In response to the 150 mM NaCl treatment, the Na^+^ concentrations in the roots and shoots increased by 5.5 and 9.8 times, respectively; the K^+^ concentrations in the roots and shoots decreased by 69.2 and 49.3%, respectively; and the Na^+^/K^+^ ratio in the roots and shoots increased by 20.4 and 21.4 times, respectively. Under non-stressed conditions, the application of DCPTA (20–400 μm) have no effect on the Na^+^ concentrations and Na^+^/K^+^ ratio in both the roots and shoots, the K^+^ concentrations in the shoots and the shoot/root Na^+^ ratio of plants, while the application of 100 μm DCPTA significantly increased the K^+^ concentrations in the roots. NaCl induced increases in Na + concentrations and decreases in K^+^ concentrations, in both the shoots and roots, and these alterations were, in general, alleviated by the used of DCPTA. One hundred micromolar (100 μM) of DCPTA appeared to be the most effective concentration for decreasing Na^+^ concentrations and increasing K^+^ concentrations under salinity stress. DCPTA application significantly affected the Na^+^/K^+^ ratio in both the roots and shoots of salinity-stressed plants. Under salinity stress, compared with those of non-DCPTA-treated plants, the Na^+^/K^+^ ratios of the shoots of plants treated with 20, 100 and 400 μM DCPTA decreased by 59.1, 68.6, and 39.7%, respectively, and the ratio of Na^+^/K^+^ in the roots decreased by 61.1, 71.3, and 22.3%, respectively. Furthermore, the NaCl-induced increases in the shoot/root Na^+^ ratio were alleviated by applications of DCPTA (20–400 μM), especially with DCPTA at 100 μM.

**Table 3:** Analysis of variance and mean comparisons for ion concentrations (K^+^ and Na^+^) in the shoots and roots of maize plants grown under two salinity levels and treated with DCPTA at four rates of application

Source of variation	Shoot			Root		
	Na^+^ content (mg·g^−1^ DW)	K^+^ content (mg·g^−1^ DW)	Na^+^/K^+^ ratio	Na^+^ content (mg·g^−1^ DW)	K^+^ content (mg·g^−1^ DW)	Na^+^/K^+^ ratio
NaCl (mM) (N)						
0	2.43 ± 0.29b	50.39 ± 3.11a	21.11 ± 0.01a	5.13 ± 0.60b	22.57 ± 2.03a	0.23 ± 0.03b
150	17.54 ± 5.15a	33.05 ± 6.67b	2.11 ± 0.27b	29.20 ± 5.36a	10.64 ± 3.79b	3.27 ± 1.64a
DCPTA (μM) (D)						
0	14.02 ± 12.30a	38.57 ± 13.43a	11.37 ± 0.50a	20.37 ± 15.87a	14.27 ± 8.04a	2.79 ± 1.73a
20	8.22 ± 6.39a	43.34 ± 8.83a	13.21 ± 0.19a	16.34 ± 11.34a	18.78 ± 6.21a	1.15 ± 0.98a
100	7.65 ± 5.51a	46.11 ± 5.80a	12.00 ± 0.14a	13.64 ± 9.73a	18.91 ± 4.42a	0.86 ± 0.72a
400	10.04 ± 7.74a	38.86 ± 10.58a	9.86 ± 0.29a	18.30 ± 14.24a	14.47 ± 7.21a	2.19 ± 1.08a
Significance						
NaCl (mM) (N)	***	***	***	***	***	***
DCPTA (μM) (D)	***	***	**	***	***	***
N × D	***	***	ns	***	***	***

ns, *, **, and ***: Not significant or significant at *P* ≤ 0.05, 0.01, and 0.001, respectively. The different letters within each column indicate significant differences according to Duncan’s multiple-range test (*P* = 0.05)

### Exogenous DCPTA maintains K^+^/Na^+^ homeostasis by altering the expression of *ZmSOS1*, *ZmHKT1*, *ZmNHX1* and *ZmSKOR*

**Fig. 8:**
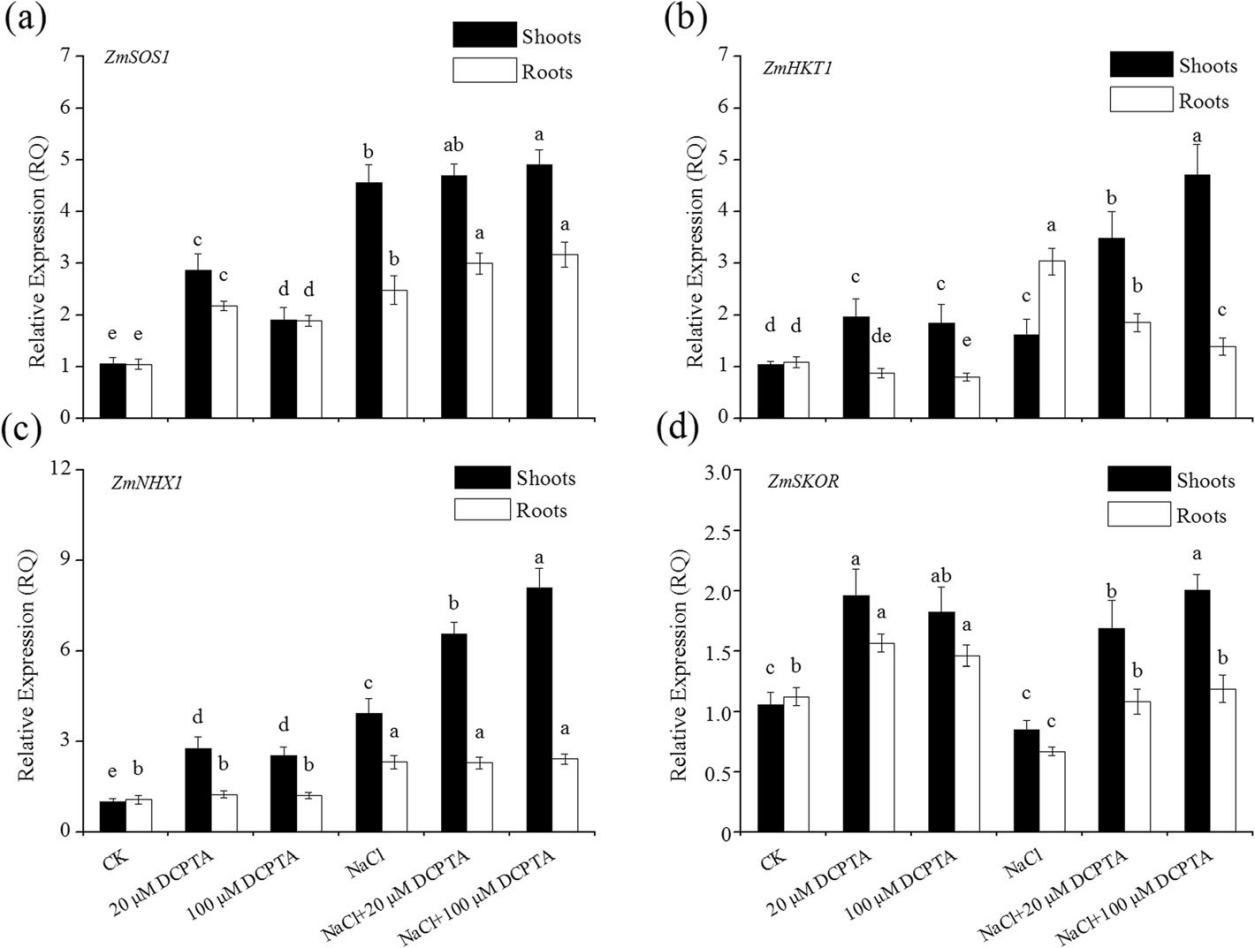
Effects of DCPTA on the expression of *ZmSOS1* (**a**), *ZmHKT1* (**b**), *ZmNHX1* (**c**), and *ZmSKOR* (**d**) in the roots and leaves of non-stressed and salinity-stressed maize seedlings. The plants were grown in a hydroponic solution that contained 0, 20, 100, or 400 μM DCPTA with or without 150 mM NaCl. The data are the means ± SEs (*n* = 5). The different letters on the bars indicate significant differences according to Duncan’s test (*P* = 0.05)

NaCl significantly affected the expression of *ZmSOS1*, *ZmNHX1* and *ZmHKT1* in both the shoots and roots and *ZmSKOR* in the roots of maize plants (*P* ≤ 0.001). DCPTA significantly affected the expression of *ZmSOS1*, *ZmNHX1*, *ZmHKT1* and *ZmSKOR* in both the shoots and roots of maize plants. And the interaction between NaCl and DCPTA significantly affected the expression of *ZmSOS1*, *ZmHKT1* in both the shoots and roots and Zm*NHX1* in the shoots of maize plants (Table 4). As shown in Fig. 8a, the expression of *ZmSOS1* was significantly upregulated in both the roots and shoots of the plants treated with 20 and 100 μM DCPTA under non-stressed conditions, and the plants treated with 100 μM DCPTA under salinity-stressed conditions. As shown in Fig. 8b, in the shoots, DCPTA application significantly increased *ZmHKT1* expression under both stressed and non-stressed conditions. In the roots, DCPTA application significantly decreased *ZmHKT1* expression under stressed conditions. Under both stressed and non-stressed conditions, the expression of *ZmNHX1* was significantly upregulated in the shoots (Fig. 8c) and the expression *ZmSKOR* was upregulated in both the roots and shoots of plants (Fig. 8d), in response to application of DCPTA at 20 and 100 μM – more markedly with the latter.

**Table 4:** Analysis of variance and mean comparisons for the expression of four genes (*ZmSOS1*, *ZmHKT1*, *ZmNHX1*, and *ZmSKOR*) responsible for Na^+^ and/or K^+^ uptake, transport and compartmentalization in the shoots and roots of maize plants grown under two salinity levels and treated with DCPTA at four rates of application

Source of variation	Relative expression (RQ) in shoots				Relative expression (RQ) in roots			
	*ZmSOS1*	*ZmNHX1*	*ZmHKT1*	*ZmSKOR*	*ZmSOS1*	*ZmNHX1*	*ZmHKT1*	*ZmSKOR*
NaCl (mM) (N)								
0	1.94 ± 0.80b	2.10 ± 0.85b	1.62 ± 0.50b	1.61 ± 0.45a	1.70 ± 0.50b	1.17 ± 0.14b	0.95 ± 0.13b	1.38 ± 0.21a
150	4.72 ± 0.31a	6.20 ± 1.85a	3.27 ± 1.39a	1.51 ± 0.53a	2.88 ± 0.37a	2.35 ± 0.19a	2.09 ± 0.74a	0.98 ± 0.25b
DCPTA (μM) (D)								
0	2.81 ± 1.86a	2.47 ± 1.58b	1.33 ± 0.37b	0.95 ± 0.14b	1.77 ± 0.78b	1.69 ± 0.68a	2.06 ± 1.05a	0.90 ± 0.25b
20	3.78 ± 1.01a	4.67 ± 2.03a	2.73 ± 0.90a	1.82 ± 0.26a	2.58 ± 0.45a	1.77 ± 0.58a	1.37 ± 0.53b	1.32 ± 0.27a
100	3.41 ± 1.60a	5.31 ± 2.97a	3.28 ± 1.58a	1.91 ± 0.19a	2.53 ± 0.70a	1.82 ± 0.66a	1.15 ± 0.29b	1.33 ± 0.17a
Significance								
NaCl (mM) (N)	***	***	***	ns	***	***	***	***
DCPTA (μM) (D)	***	***	***	***	***	***	***	***
N × D	**	***	***	ns	*	ns	***	ns

ns, *, **, and ***: Not significant or significant at *P* ≤ 0.05, 0.01, and 0.001, respectively. The different letters within each column indicate significant differences according to Duncan’s multiple-range test (*P* = 0.05)

## Discussion

The harmful effects caused by salinity stress involve various physiological and biochemical mechanisms related to plant growth and development [4, 42]. Biomass is a reliable indicator of plant responses to stress, and one of the primary effects of salinity stress is the inhibition of plant growth [4]. In this study, salinity stress significantly reduced the growth of maize seedlings, as indicated by the decreased DW of the roots and shoots of maize seedlings. However, the growth inhibition under salinity stress was alleviated by the application of DCPTA, particularly at 100 μM. Research has shown that there is a two-phase growth response to salinity: the growth is first reduced by the decrease in soil water potential and then by salt injury to the older leaves, because of a rapid rise in salt concentration in cell walls of cytoplasm when the vacuoles can no longer sequester incoming salts [4]. The promotion of DCPTA on the growth of maize seedlings under salinity stress may be related to the maintained water status and K^+^/Na^+^ homeostasis. The beneficial influence of DCPTA on biomass production has also been reported for both soybean [43] and maize [35] under drought stress and for maize under low-temperature stress [33, 36].

The reduction in plant growth under salinity conditions is due to the detrimental effect on bioenergetic processes of photosynthesis caused by salinity [1]. In this study, NaCl stress reduced the P_n_ in maize seedlings. However, exogenous DCPTA (20 and 100 μM) alleviated NaCl-induced inhibition in photosynthesis, which correlated with the improved biomass production in the shoots and roots of plants treated with DCPTA exposed to salinity conditions. Stomatal closure in response to decreases in leaf turgor to minimize water loss is an important response of plants to salinity stress, which is accompanied by notable declines in g_s_, consequently, limited ambient CO_2_ diffusion to the site of carboxylation and stomatal limitation of photosynthesis [13]. Consistently, we also observed that g_s_ significantly decreased in response to NaCl stress, and accompanied by a decrease in T_r_. However, these reduction were alleviated by DCPTA (20 and 100 μM), which is consistent with the results of a previous study by Xie et al. [35] in which DCPTA exerted positive effects on plant photosynthetic capability. The increased g_s_ and T_r_ may be related to the maintained water status and the enhanced root water absorption capacity in DCPTA-treated plants, and thus reduce the stomatal limitation of photosynthesis [35]. In addition, salinity stress significantly increased the C_i_, which may be due to decreased CO_2_ assimilation induced by photosystem photo-oxidation or damage and the inactivation of the photosynthesis enzymes under salinity stress [13]. Such results indicate that non-stomatal limitations were the primary cause of the decrease in the P_n_ of plants grown under salinity conditions [44]. However, the increase in C_i_ induced by salinity stress were alleviated by 100 μM DCPTA, and the decreased C_i_ in DCPTA-treated plants may be related to the enhanced CO_2_ assimilation capacity, which depends on the integrity of photosynthetic organ structure and function, and thus reduce the non-stomatal limitation of photosynthesis in DCPTA-treated plants [4]. The alleviation of stomatal and non-stomatal limitation by DCPTA contributed to improvements in photosynthetic capacity under salinity stress.

Chlorophylls are essential pigments that capture light energy and perform photosynthesis in plants. *F*_v_/*F*_m_, ΦPSII and qP are parameters that reflect photochemical quenching, whereas NPQ reflects non-photochemical quenching [45]. In the current study, NaCl stress decreased the values of the chlorophyll contents, *F*_v_/*F*_m_, ΦPSII and qP, and these reductions were alleviated with the application of DCPTA at 20 μM and 100 μM. This suggests that exogenous DCPTA may enhance the ability to absorb and transform light energy and improve the quantum efficiency of PSII photochemistry, which would lead to enhanced photosynthesis under salinity conditions. In addition, exogenous DCPTA alleviated the salinity-induced decrease in qP and increase in NPQ, which indicated that DCPTA could promote the use efficiency of absorbed light in photochemical processes, with minimal thermal dissipation and fluorescence emissions [33, 35]. This is supported by the observation that DCPTA improved P_n_ in maize seedlings under salinity stress. The increase of chlorophyll content and the improvement in PSII efficiency may be related to a promising role of DCPTA in protecting photosynthetic apparatus from oxidative damage under stress conditions [35, 46]. Thus, these results indicated that, DCPTA improved PSII efficiency, which ultimately increased the photosynthetic capability and salinity tolerance of maize.

The promotion of increased plant biomass and photosynthetic capacity due to the effects of DCPTA can also be attributed to an amelioration of water status. In this study, the application of 20 μM and 100 μM DCPTA alleviated the decrease in water status under salinity stress, as indicated by the LRWC and leaf Ψω, which may be attributed to the dynamic balance maintained between plant root water absorption and leaf transpiration [13]. Moreover, the relatively low reduction in the LRWC and leaf Ψω in the stressed plants treated with DCPTA was consistent with the relatively low reduction in g_s_ and T_r_ in those plants. Given that the beneficial effect of DCPTA on plant water status is due to an increase in root water absorption, which is largely achieved by improved root growth (which is associated with the root DW, length, surface area and volume) and Lp, not a decrease in water loss due to the increase in g_s_ and T_r_. These results suggest that DCPTA increased the root water uptake ability and thus improved the water status of maize plants under salinity stress.

Salts absorbed by the roots are transported to the shoots over long distances in the transpiration stream, and leaves are the main location of Na^+^ accumulation in most plants [4]. In this study, the addition of DCPTA induced substantial decreases in the Na^+^ concentration in the shoots, which was accompanied by a decreased shoot/root Na^+^ ratio under salinity stress. To reduce cytoplasmic Na^+^ concentrations, plants have evolved various adaptive mechanisms, including restricting Na^+^ uptake from the soil solution, extruding excessive Na^+^ and vacuolar partitioning of Na^+^ to decrease Na^+^ accumulation in the cytosol [47]. In this study, under salinity stress conditions, the expression of *ZmSOS1* in the roots and shoots was upregulated, and DCPTA further upregulated the expression of *ZmSOS1* in the roots; in turn, this upregulation decreased the Na^+^ influx from the external solution into the cytosol or promoted Na^+^ export to the apoplastic space [11, 48]. Therefore, DCPTA may increase the capability of plants to extrude Na^+^ into soil solution and/or mitigate the toxic effects of Na^+^, as the toxicity of Na^+^ is small in the apoplastic space [49]. DCPTA increased shoot *ZmHKT1* expression under salinity stress, which facilitated Na^+^ recirculation into the xylem and Na^+^ allocation to the roots. In contrast, DCPTA decreased *ZmHKT1* expression in the roots, which reduced Na^+^ loading into the xylem and subsequently transported Na^+^ to sensitive photosynthetic tissues, suggesting that, compared with untreated plants, plants treated with DCPTA may be more capable of restricting Na^+^ accumulation in sensitive tissues. *HKT* transporters have been shown to mediate the translocation of Na^+^ from the roots to the shoots by retrieving Na^+^ from the root-to-shoot xylem sap, which represents a strategy to avoid the toxic effects of the photosynthetic apparatus [9, 42]. In this study, the dysregulation of *ZmHKT1* under salinity stress and in response to DCPTA was correlated with the lower shoot/root Na^+^ ratio in the plants treated with DCPTA compared with the untreated plants. Increasing the expression of *ZmSOS1* in the roots and *ZmHKT1* in the shoots while decreasing the expression of *ZmHKT1* in the roots contributed to decreased Na^+^ concentration in the shoots of the plants treated with DCPTA and could therefore be a key consequence of the effect of DCPTA on plants exposed to salinity stress. In addition, a recent study showed that Na-acclimated maize plants have improved vacuolar Na^+^ sequestration ability in their leaves and can accumulate relatively large amounts of Na^+^ in those organs without any detrimental effects on photosynthetic capacity [50]. It has been proposed that *NHX* functions in Na^+^ compartmentalization in the vacuole and efflux of Na^+^ from cells [4], and the upregulation of *NHX* in transgenic plant species such as *Brassica napus* [51], poplar [52] and tomato [53] has been shown to increase plant salinity tolerance. Moreover, a previous study showed that salinity-tolerant maize hybrids exhibited higher expression of *ZmNHX1* than did salinity-sensitive hybrids exposed to salinity stress [54]. In this study, *ZmNHX1* expression was upregulated in both roots and shoots of plants under salinity stress, and further upregulated in the shoots of DCPTA-treated plants under salinity conditions. The upregulation of *ZmNHX1* expression in the shoots in response to DCPTA was responsible for increased Na^+^ compartmentalization in the leaf vacuoles, which was beneficial for reducing the Na^+^ toxicity in the cytosol, improving the osmolarity in the vacuole, and concomitantly enhancing plant salinity tolerance [55].

Owing to the similar hydration radius of K^+^ and Na^+^, excess Na^+^ osmoticum obviously competes for K^+^ entry into the symplast at transport sites, which causes a reduction in K^+^ concentration and alters the Na^+^/K^+^ ratio under salinity stress [18]. In this study, DCPTA significantly increased the K^+^ concentration and decreased the Na^+^/K^+^ ratio in the plants under salinity stress, which was presumably crucial for salinity tolerance [4]. It has been shown that the *SKOR* channel influences the xylem loading of K^+^ [16]. In this study, DCPTA significantly upregulated the expression of *ZmSKOR* in plants exposed to 150 mM NaCl, which may have contributed to K^+^ release into the xylem for transport towards the shoots [22, 56] and was correlated with increased K^+^ concentration in plants treated with DCPTA in this study. Moreover, the upregulation of *ZmSKOR* in combination with *ZmSOS1* and *ZmHKT1* in the shoots as well as the downregulation of *ZmHKT1* in the roots of plants treated with DCPTA can account for the reduced Na^+^/ K^+^ ratio of plants under salinity stress.

## Conclusions

**Fig. 9:**
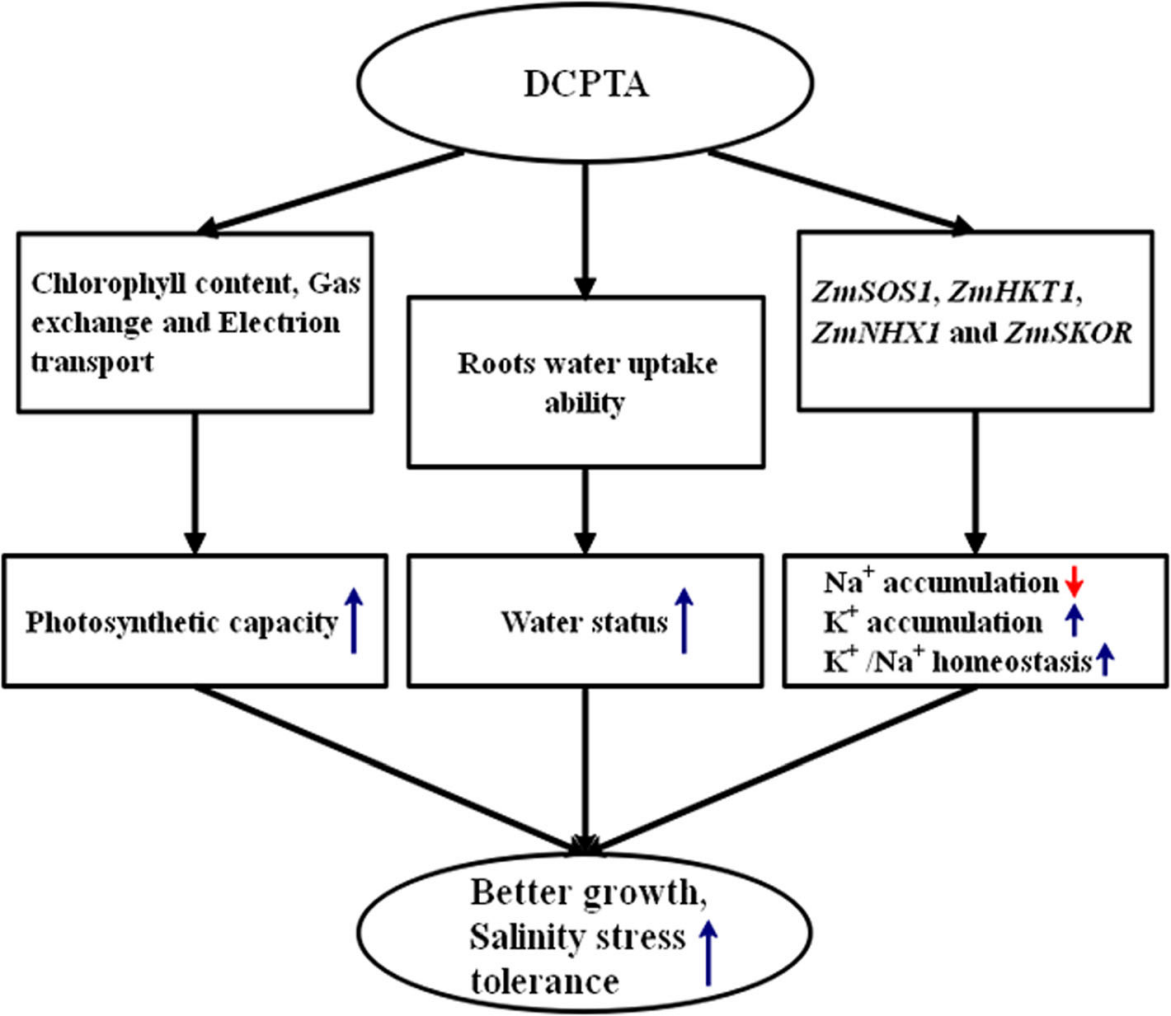
Schematic representation of the positive role of DCPTA on the salinity tolerance of maize. A model was developed to show that the photosynthetic capacity, water status, accumulation of Na^+^ and K^+^ and the Na^+^/K^+^ ratio were regulated by DCPTA in maize under salinity stress. The blue arrows (↑) and the red arrows (↓) represent the positive and passive roles of DCPTA, respectively

In conclusion, we have demonstrated that 100 μM DCPTA can enhance the salinity stress tolerance of maize seedlings. DCPTA improved the photosynthetic capacity of maize plants by regulating stomatal movement and improving both light energy absorption and electron transport in PSII; DCPTA maintained the water status by promoting root growth and improving root water uptake ability, which contributed to water transport to specific tissues and CO_2_ diffusion across the plasma membrane; In addition, DCPTA pre-treatment decreased the Na^+^ concentration and increased the K^+^ concentration by altering the expression of four genes (*ZmSOS1*, *ZmHKT1*, *ZmNHX1* and *ZmSKOR*) that encode membrane transport proteins responsible for K^+^/Na^+^ homeostasis under salinity stress. The improved photosynthesis, water status, and K^+^/Na^+^ homeostasis contributed to improved growth and salinity stress tolerance (Fig. 9). Thus, applications of appropriate concentration DCPTA can be a sustainable approach to increase crop yields under salinity stress.

## Methods

### Plant growth conditions, stress treatments and sampling

Seeds of maize (cultivar ZD958, a local commonly planted cultivar) (Beijing De Nong Seed Industry Co., Ltd., Beijing, China) were surface sterilized for 10 min with mercuric chloride (0.2%) and then rinsed abundantly with distilled water. Afterward, the seeds were soaked in distilled water for 12 h and then germinated on double-layered filter paper moistened with distilled water at 28 °C for 2 days in the dark. The germinated seeds were sown in quartz sand in a climate chamber (HPG-280HX, Harbin Donglian Electronic Technology Development Co., Ltd., Harbin, China) whose temperature was set to 28 °C/18 °C, corresponding to a 12 h/12 h (day/night) photoperiod; the light intensity in the chamber was 350–450 μmol·m^− 2^·s^− 1^, and the relative humidity was 50–60%. Two-leaf-stage seedlings of uniform size were transferred to plastic pots (40.5 cm × 32.5 cm × 12 cm, 12 plants per pot) containing 1/2-strength Hoagland nutrient solution (pH 6.5). The nutrient solution was changed every 3 days to avoid nutrient depletion. The nutrient solution comprised the following components: 2.5 mM Ca (NO_3_)_2_, 1.0 mM K_2_SO_4_, 0.2 mM KH_2_PO_4_, 0.6 mM MgSO_4_, 2.5 mM CaCl_2_, 0.5 mM NaCl, 1.0 μM H_3_BO_4_, 2.0 μM MnSO_4_, 0.5 μM ZnSO_4_, 0.3 μM CuSO_4_, 0.005 μM (NH_4_)_6_Mo_7_O_24_, and 200 μM Fe-EDTA. When the seedlings grew to the three-leaf stage, they were treated with the following different nutrient solutions: (1) 1/2-strength Hoagland nutrient solution (CK); (2) 1/2-strength Hoagland nutrient solution + 20 μM DCPTA (20 μM DCPTA); (3) 1/2-strength Hoagland nutrient solution + 100 μM DCPTA (100 μM DCPTA); (4) 1/2-strength Hoagland nutrient solution + 400 μM DCPTA (400 μM DCPTA); (5) 150 mM NaCl in 1/2-strength Hoagland nutrient solution + 0 μM DCPTA (NaCl); (6) 150 mM NaCl in 1/2-strength Hoagland nutrient solution + 20 μM DCPTA (NaCl + 20 μM DCPTA); (7) 150 mM NaCl in 1/2-strength Hoagland nutrient solution + 100 μM DCPTA (NaCl + 100 μM DCPTA); and (8) 150 mM NaCl in 1/2-strength Hoagland nutrient solution + 400 μM DCPTA (NaCl + 400 μM DCPTA). The DCPTA (Zhengzhou Zhengshi Chemical Limited Co., Ltd., China) was applied 2 days before the addition of NaCl in the nutrient solution. The NaCl concentration was gradually increased in 50 mM increments every 8 h to avoid salinity shock. The experiment included 8 treatments with 5 replicates (90 plants in total per treatment). Each replicate included 18 plants (selected from 2 pots of 12 plants each). Five days after the salinity level reached 150 mM, the gas exchange, chlorophyll fluorescence parameters, LRWC and leaf Ψω were measured for the second fully expanded leaf, and the root Lp was measured. The plants in the 8 treatments were then collected, frozen immediately in liquid nitrogen and stored at − 80 °C until the plant biomass determination and biochemical assays.

In the second experiment, the plants were cultivated in the same manner as described above. After 24 h of salinity treatment (0 and 150 mM NaCl) in combination with DCPTA (0, 20 and 100 μM), the leaves and roots of the maize seedlings of 6 treatments (including five replicates) were sampled by mixing 5 seedlings per replicate, frozen immediately in liquid nitrogen and then stored at − 80 °C for RT-qPCR .

### Analysis of plant growth, root characteristic parameters and ion concentrations

After 5 days of the treatments, the plants were divided into two parts: roots and shoots. The root characteristic parameters, including the root length, surface area, and volume, were then scanned with a root scanner (MRS-9600TFU2L, Shanghai Microtek Technology Co., Ltd. Shanghai, China). The roots and shoots were subsequently dried in an oven at 80 °C to a constant weight to determine the DW. The dry samples of the roots and shoots were ground into a fine powder and passed through a 1 mm diameter mesh stainless steel sieve. Afterward, 0.1 g of the root and shoot samples was digested with a 5 mL of a HNO_3_:HClO_4_ (5:1 v/v) solution at 80 °C until the sample consisted of only a small white residue. The sample was then brought to 50 mL with deionized water. The contents of K^+^ and Na^+^ in the roots and shoots were analysed by flame atomic absorption spectrophotometry (Z-2000; Hitachi, Japan).

### Determination of LRWC

To analyse the LRWC, the second fully expanded leaves were collected and weighed to determine the fresh weight (FW), after which they were immersed in distilled water for 24 h in the dark. The leaves were subsequently gently blotted dry with absorbent paper and weighed to determine their turgid weight (TW). The samples were then dried in an oven at 105 °C for 20 min followed by 80 °C until a constant weight was achieved, at which point the DW was determined. The LRWC was calculated using the following formula: LRWC (%) = [(FW - DW) / (TW - DW)] × 100.

### Measurement of leaf Ψω

The leaf Ψω of the second fully expanded leaves was determined by the pressure chamber technique (type 3115 pressure chamber, Beijing Huahai Heng Hui Technology Co., Ltd. Beijing, China). The second fully expanded leaves were harvested and inserted immediately into the rubber plug hole of the pressure chamber such that the incision was exposed approximately a few millimetres outside the sealing ring for convenient observation. After a good seal was confirmed, the pressure control valve was rotated to slowly pressurize at 0.05 MPa·s^− 1^. When a water film appeared at the incision, rotating of the control valve was stopped, and the pressure value at this time was recorded as the leaf Ψω.

### Measurement of chlorophyll content

To measure the chlorophyll content in the leaves, a fresh leaf sample (0.5 g) and 5 mL of acetone (80% v/v) were homogenized together in an ice bath. After centrifugation at 10,000 g for 10 min at 4 °C, the absorbance at 645 and 663 nm was monitored spectrophotometrically with a spectrophotometer (UV-5500, Shanghai Yuan Analysis Instrument Co., Ltd., China) to determine the contents of chlorophyll a (Chl a) and chlorophyll b (Chl b), respectively. These contents were calculated in accordance with the equations of Arnon [57].

### Determination of gas exchange parameters

The gas exchange of attached leaves were determined with an LI-6400 portable photosynthetic system (LI-COR Inc., USA) at 9:00–11:00 a.m. for the different treatments of maize seedlings. The second fully expanded leaves were used for the assays. During the measurements, the leaf chamber temperature was maintained at approximately 26 °C, the photosynthetic photon flux density (PPFD) was 800 μmol m^− 2^ s^− 1^, the CO_2_ concentration was 400 μmol·mol^− 1^, and the relative humidity was 60–70%. The gas exchange parameters P_n_, g_s_, C_i_ and T_r_ were recorded simultaneously with five seedlings per treatment.

### Determination of chlorophyll fluorescence parameters

Chlorophyll fluorescence was determined via a PAM2000 modulated fluorescence spectrometer (Walz, Germany). After 30 min of dark adaptation, the minimum fluorescence (*F*_o_) was obtained by irradiating the measured light (< 0.05 μmol·m^− 2^·s^− 1^), and the maximum fluorescence (*F*_m_) was measured via a saturated pulse light (0.8 s; 8000 μmol·m^− 2^·s^− 1^). The photosynthetic steady-state fluorescence (*F*_s_) was measured by turning on the actinic light (1 h; 300 μmol·m^− 2^·s^− 1^), the saturated pulse light (8000 μmol·m^− 2^·s^− 1^) was turned on again to obtain the maximum fluorescence (*F*_m_′), the actinic light was turned off, the far-red light was turned on immediately, and the minimum fluorescence (*F*_o_’) under the light was obtained. Other parameters were calculated as follows: the *F*_v_/*F*_m_ = (*F*_m_ - *F*_o_) / F_m_; the photochemical quantum efficiency of PSII (ΦPSII) = (*F*_m_′ – *F*_s_) /Fm′; the photochemical quenching coefficient (qP) = (*F*_m_′- *F*_s_) / (*F*_m_′- *F*_o_’); and the non-photochemical quenching (NPQ) coefficient = (*F*_m_ – *F*_m_′) /*F*_m_′ [45].

### Measurement of root Lp

The determination of Lp was performed according to the method of Nardini et al. [58], with slight modifications. The roots of 5 maize seedlings per treatment were cut and removed. The whole roots were then placed in a pressure chamber, the cut was exposed to the outside of the seal ring by approximately a few centimetres, and a gradually increasing pressure (from 0.1 to 0.4 MPa) was applied to the roots. The sap was collected with pre-prepared, dried absorbent cotton in Eppendorf tubes at each pressure gradient (for 1 min) and then weighed on a precision balance, thereby generating a range of sap flows that represented the whole-plant T_r_. The root Lp was calculated according to the following formula: Lp = Jv/P, where Jv (m·s^− 1^) is the flow rate and P (MPa) is the external pressure.

### RNA extraction and quantitative RT-qPCR

To extract RNA using a Qiagen RNeasy Plant Mini Kit (Shanghai Baili Biotechnology Co., Ltd., Shanghai, China), 0.1 g of root and shoot samples was used. The cDNA was then reverse transcribed using an iScript™ cDNA synthesis kit (Bio-Rad, California, USA) kit. The cDNA was diluted 50-fold, and 2 μL was taken for qRT-PCR analysis. Actin1 was used as an internal reference gene. A relative quantitative analysis was performed using the 2^-△△CT^ method. The primers used are listed in Table S1. Five replicates per treatment and 5 technical replicates per plant were included.

### Statistical analysis

SPSS 19.0 (IBM, Armonk, NY, USA) was used for statistical analysis. The data were subjected to two-way ANOVA with three sources of variation (NaCl, DCPTA and their interaction), and the differences between the treatment means within each measured parameter were compared via Duncan’s multiple range test (*P* < 0.05) (Additional file 2).

## Supplementary information

**Additional file 1: Table S1.** List of primer sequences for qPCR analysis.

**Additional file 2.** Research data.

## Data Availability

The datasets used and/or analysed during the current study are available from the corresponding author on reasonable request.

## Abbreviations

C_i_: intracellular CO_2_ concentration
Chl a: chlorophyll a
Chl b: chlorophyll b
DCPTA: 2-(3, 4-dichlorophenoxy) triethylamine
DW: dry weight
*F*_o_: minimum fluorescence
*F*_m_: maximal fluorescence yield of the dark-adapted state
*F*_v_/*F*_m_: maximal quantum yield of PSII photochemistry
g_s_: stomatal conductance
Jv: sap flow
Lp: root hydraulic conductivity
LRWC: leaf relative water content
NPQ: non-photochemical quenching
P: pressure
P_n_: net photosynthesis rate
PSII: photosystem II
PPFD: photosynthetic photon flux density
qP: photochemical quenching coefficient
ROS: reactive oxygen species
T_r_: transpiration rate
Ψω: water potential
ΦPSII: effective quantum yield of PSII photochemistry
