# Supplementary material for: Exogenous 2-(3,4-Dichlorophenoxy) triethylamine alleviates salinity stress in maize by enhancing photosynthetic capacity, improving water status and maintaining K+/Na+ homeostasis
Source: BMC Plant Biol. 2020 Jul 23;20:348. doi: 10.1186/s12870-020-02550-w (PMC7376668; doi:10.1186/s12870-020-02550-w)
Supplement: Supplementary file 1 — Additional file 1: Table S1. List of primer sequences for qPCR analysis. [file 12870_2020_2550_MOESM1_ESM.docx]

**Table S1.** List of primer sequences for qPCR analysis

| Gene | Forward primer sequence (5′ to 3′) | Reverse primer sequence (5′ to 3′) |
| --- | --- | --- |
| *ZmSOS1* | GCTTGTCACATACTTCACAG | ACTTGTCCACTTCACTACAC |
| *ZmHKT1* | TCGGCTCTGGACCTACTCTT | ACGACGACGACTCTGCTCTA |
| *ZmNHX1* | ATGCAGGGTTCCAAGTGAAG | AATATTGCCCCAAGTGCAAG |
| *ZmSKOR* | TCAGATCCAAGATGTCCCAG | TTCGTATCCTCTTAACGCAG |
| Actin1 | GACCTCACCGACCACCTAATG | CTGAACCTTTCTGACCCAATG |
